# Tiotropium might improve survival in subjects with COPD at high risk of mortality

**DOI:** 10.1186/1465-9921-15-64

**Published:** 2014-06-10

**Authors:** Pierre-Régis Burgel, Jean-Louis Paillasseur, Daniel Dusser, Nicolas Roche, Dacheng Liu, Yufeng Liu, Armin Furtwaengler, Norbert Metzdorf, Marc Decramer

**Affiliations:** 1 Hôpitaux Universitaires Paris Centre, Assistance Publique-Hôpitaux de Paris, Paris, France; 2 Université Paris Descartes, Sorbonne Paris Cité, Paris, France; 3 Clindatafirst, Clamart, France; 4 Boehringer Ingelheim Pharma GmbH & Co KG, Ridgefield, CT, USA; 5 University of North Carolina at Chapel Hill, Chapel Hill, North Carolina, USA; 6 Boehringer Ingelheim Pharma GmbH & Co KG, Ingelheim, Germany; 7 Respiratory Division, Katholieke Universiteit Leuven, University Hospital, Leuven, KU, Belgium

**Keywords:** Mortality, COPD, Exacerbations, Tiotropium

## Abstract

**Background:**

Inhaled therapies reduce risk of chronic obstructive pulmonary disease (COPD) exacerbations, but their effect on mortality is less well established. We hypothesized that heterogeneity in baseline mortality risk influenced the results of drug trials assessing mortality in COPD.

**Methods:**

The 5706 patients with COPD from the Understanding Potential Long-term Impacts on Function with Tiotropium (UPLIFT®) study that had complete clinical information for variables associated with mortality (age, forced expiratory volume in 1 s, St George’s Respiratory Questionnaire, pack-years and body mass index) were classified by cluster analysis. Baseline risk of mortality between clusters, and impact of tiotropium were evaluated during the 4-yr follow up.

**Results:**

Four clusters were identified, including low-risk (low mortality rate) patients (n = 2339; 41%; cluster 2), and high-risk patients (n = 1022; 18%; cluster 3), who had a 2.6- and a six-fold increase in all-cause and respiratory mortality compared with cluster 2, respectively. Tiotropium reduced exacerbations in all clusters, and reduced hospitalizations in high-risk patients (p < 0.05). The beneficial effect of tiotropium on all-cause mortality in the overall population (hazard ratio, 0.87; 95% confidence interval, 0.75–1.00, p = 0.054) was explained by a 21% reduction in cluster 3 (p = 0.07), with no effect in other clusters.

**Conclusions:**

Large variations in baseline risks of mortality existed among patients in the UPLIFT® study. Inclusion of numerous low-risk patients may have reduced the ability to show beneficial effect on mortality. Future clinical trials should consider selective inclusion of high-risk patients.

## Background

Chronic obstructive pulmonary disease (COPD) is a major health problem associated with disability, healthcare utilization, and premature death [1]. Clinical trials have established that current pharmacologic approaches can improve lung function and reduce exacerbations, but their efficacy in modifying the disease course is a subject of debate [2]. In recent years, large long-term clinical trials evaluated pharmacologic intervention on mortality rates (as primary or secondary outcomes) in patients with COPD [3,4]. Although these studies have suggested that current inhaled therapies have some effects on mortality [3,5], no study has shown unequivocally that current pharmacotherapy reduced mortality in patients with COPD [6].

One possible explanation for the limited effect seen in long-term clinical trials on mortality [3,5] could be an actually limited efficacy of current drugs on this outcome. Alternatively, these results may have been related to the design of clinical trials that assessed mortality in patients with COPD, and the heterogeneity of baseline mortality risk among those patients recruited in these clinical trials may have played an important role: it is well established that most trial outcomes (e.g. mortality) occur in a relatively small number of high-risk patients, while most patients are at much lower risk [7,8]. A clinical trial enrolling a large group of patients with COPD at low risk of outcome may find the therapy useless overall, but miss detecting its efficacy in high-risk patients [7]. These considerations suggest the importance of risk stratification in clinical trials [7]. However, stratification of mortality risks was not performed in the recent Towards a Revolution in COPD Health (TORCH) [3] and Understanding Potential Long-term Impacts on Function with Tiotropium (UPLIFT®) [4] clinical trials, which may have limited their ability to detect important differences in mortality.

In the present study, we explored the possibility that the population recruited in the UPLIFT® study, a 4-year trial of tiotropium use in patients with COPD, was composed of patients with large variations in baseline mortality risk. Our strategy was to examine mortality risk heterogeneity using a cluster analysis of variables selected for their previously reported association with increased mortality, and obtained at recruitment in the study. Our goals were to identify clusters of patients with different mortality risks at baseline and to examine the impact of risk stratification on the evaluation of tiotropium effect on mortality, as well as to an outcome associated with increased mortality, e.g. exacerbations. Some of the results of these studies have been previously reported in abstract form [9].

## Methods

### Patients and study design

UPLIFT® was a 4-year, randomized, placebo-controlled, clinical trial in 5993 patients with COPD. Details of the study design and results have been previously reported [4,10]. All patients gave written informed consent and the study was approved by a local Institutional Review Board (IRB) or an Independent Ethics Committee (IEC) at each center prior to the start of the study. The constitution of the IRB or IEC met the requirements of each of the 37 participating countries. Criteria for participation included diagnosis of COPD, aged at least 40 years, smoking history of at least 10 pack-years, and post-bronchodilator forced expiratory volume in 1 s (FEV_1_) ≤ 70% of the predicted normal, and FEV_1_ ≤ 70% of forced vital capacity (FVC). Post-randomization clinic visits occurred at 1 and 3 months, and then every 3 months throughout the 4-year treatment period. Eligible patients were randomized to receive either tiotropium 18 μg or a matching placebo, once daily, delivered via the HandiHaler® device (Boehringer Ingelheim Pharma GmbH & Co KG, Ingelheim, Germany).

The primary endpoints were yearly rate of decline in pre- and post-bronchodilator lung function until completion of the double-blind treatment. Secondary outcomes included other lung function measures, health-related quality of life (HRQoL) as measured by the St George’s Respiratory Questionnaire (SGRQ) total score, COPD exacerbations and related hospitalizations (severe exacerbations), and mortality. Exacerbations were defined as an increase in, or new onset of, more than one respiratory symptom with duration of 3 or more days and requiring treatment with an antibiotic and/or systemic steroid. Data from COPD exacerbations and related hospitalizations were collected on study-specific case report forms at every visit. Detailed description of mortality data collection was previously described [5]. Mortality was described at Day 1440 (protocol-defined end of 4-year treatment period) and specific causes of mortality were determined by an independent mortality adjudication committee [11]. Comorbidities were assessed based on patient’s files and self-reports.

### Statistics

#### General statistics

Data are reported as median (interquartile range) or percentage, unless otherwise specified. Statistical significance was considered at p < 0.05. For time to first exacerbation, time to first hospitalization, and time to all-cause and respiratory death, survival probability was estimated using Kaplan-Meier analysis. Cox regression with cluster was used in the control group to test cluster effect. Hazard ratio between tiotropium and placebo with each cluster were estimated using Cox regression with treatment, cluster, and treatment by cluster interaction as factors. Number of exacerbations and related hospitalizations were analyzed using a Poisson regression with over-dispersion adjusted for treatment exposure. All analyses were performed using SAS 9.2 statistical software (SAS Institute Inc, Cary, NC, USA).

#### Cluster analysis

The following clinical variables obtained at enrolment in the study were used in the cluster analysis: age [12], body mass index (BMI) [13], post-bronchodilator FEV_1_ (percent predicted) [13], smoking (pack-years) [14,15], and SGRQ total score [16]. This selection of variables was based on their previously reported association with mortality in patients with COPD, and their availability in the UPLIFT® study.

All patients with complete data for these five variables were classified using a Ward’s cluster analysis in which grouping was based on quantitative measures of similarity procedure (minimum within cluster sum of square). Pseudo-F and pseudo-t2 statistics were used to determine the optimal number of clusters in the data [17]. Relatively large pseudo F values were considered to indicate a stopping point. For the pseudo t2 statistic, we moved down the column until we found the first value markedly larger than the previous value and moved back up the column by one cluster. Clinical endpoints were analyzed for each cluster to evaluate the relevance of the clustering.

## Results

### Baseline characteristics and patient classification

**Figure 1:**
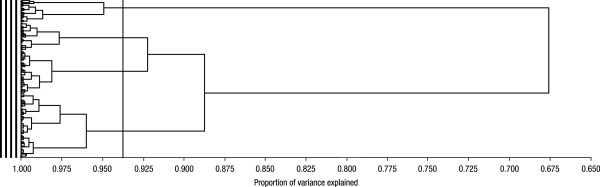
**Dendrogram showing progressive joining of the clustering process.** Data can be optimally grouped into four clusters. Characteristics of subjects in each cluster are presented in Table 1. The vertical line identifies the optimal number of clusters in the data.

The present analysis is based on 5706 patients with COPD (95.2% of the randomized population) with complete data for age, BMI, post-bronchodilator FEV_1_ (percent predicted), SGRQ total score, and cumulative smoking (pack-years). Classification of these patients using cluster analysis resulted in a dendrogram that showed the progressive joining of the clustering process (Figure 1); patients could be optimally classified in four clusters (see Methods section).

Characteristics of patients within these four clusters are presented in Table 1. Cluster 1 (n = 820 patients [14%]) contained Global Initiative for Chronic Obstructive Lung Disease (GOLD) Stage 2 or 3 patients, who were heavy smokers and had relatively preserved HRQoL, but high rates of comorbidities. Cluster 2 (n = 2339 patients [41%]) contained mostly GOLD Stage 2 patients with moderate HRQoL impairment and very low rates of comorbidities. Cluster 3 (n = 1022 patients [18%]) contained 81% of GOLD Stage 3 and 4 patients, with severe HRQoL impairment, high pack-years, and high rates of comorbidities. Compared with cluster 3, cluster 4 (n = 1525 [27%]) contained patients with less severe airflow limitation, slightly less severe HRQoL impairment, and fewer pack-years and comorbidities. Numbers of patients in the control versus tiotropium group were rather similar within each cluster (Table 1) and comparison of the main clinical characteristics of control versus tiotropium patients within each cluster showed no significant difference (not shown).

**Table 1:** Baseline characteristics of 5706 patients with COPD in the UPLIFT® trial by clusters

	**Cluster 1**	**Cluster 2**	**Cluster 3**	**Cluster 4**
	**n = 820**	**n = 2339**	**n = 1022**	**n = 1525**
**Variables used in the cluster analysis**				
Age (yrs)	66 (61–71)	67 (60–72)	63 (58–70)	63 (56–70)
BMI (kg/m^2^)	27 (24–30)	25 (23–28)	25 (22–29)	25 (22–29)
Smoking history (pack-years)	92 (80–110)	40 (30–50)	60 (47–70)	30 (20–35)
Post-bronchodilator FEV_1_ (% predicted)	49 (41–58)	55 (47–62)	36 (29–46)	43 (36–51)
SGRQ total score	40 (31–52)	34 (26–43)	60 (51–70)	55 (47–64)
**Other variables**				
Gender, M/F (%)	86/14	75/25	76/23	65/35
Current smokers (%)	28	29	34	29
Post-bronchodilator FEV_1_/FVC (%)	43 (36–51)	46 (40–54)	36 (30–45)	40 (34–48)
COPD duration (yrs)	8 (4–13)	8 (4–13)	8 (5–14)	9 (5–14)
Spirometric GOLD stage, n (% cluster) (% GOLD stage)				
GOLD stage 1	0	3 (0.1%)	0	0
GOLD stage 2	408 (50%) (16%)	1,616 (69%) (61%)	192 (19%) (7%)	432 (28%) (16%)
GOLD stage 3	372 (45%) (14%)	685 (29%) (27%)	561 (55%) (22%)	941 (62%) (36%)
GOLD stage 4	40 (5%) (8%)	35 (2%) (7%)	269 (26%) (54%)	152 (10%) (31%)
Baseline medications (%)				
LABA	54	57	62	65
ICS	54	60	65	66
ICS + LABA	43	47	51	54
Anticholinergic*	48	40	55	50
Comorbidities (%)				
Coronary artery disease	19	6	15	10
Left heart failure	4	2	4	2
Hypertension	47	16	38	25
Diabetes	13	5	10	7
**Randomization group**				
Tiotropium/control (n)	410/410	1176/1163	517/505	741/784

*Short-acting.

*BMI* = Body mass index, *COPD* = Chronic obstructive pulmonary disease, *FEV*_*1*_ = Forced expiratory volume in 1 s, *FVC* = Forced vital capacity, *GOLD* = Global Initiative for Chronic Obstructive Lung Disease, *ICS* = Inhaled corticosteroid, *LABA* = Long-acting β_2_-agonist, *SGRQ* = St George’s Respiratory Questionnaire; *UPLIFT*® = Understanding Potential Long-term Impacts on Function with Tiotropium.

### Heterogeneity in baseline risks of mortality and exacerbations by clusters in the control group

To gain knowledge on the baseline risk of mortality and exacerbations in each cluster without interference of an intervention (tiotropium), we examined these outcomes over the 4 years of the study in patients randomized in the control group (n = 2862 patients) according to clusters.

**Figure 2:**
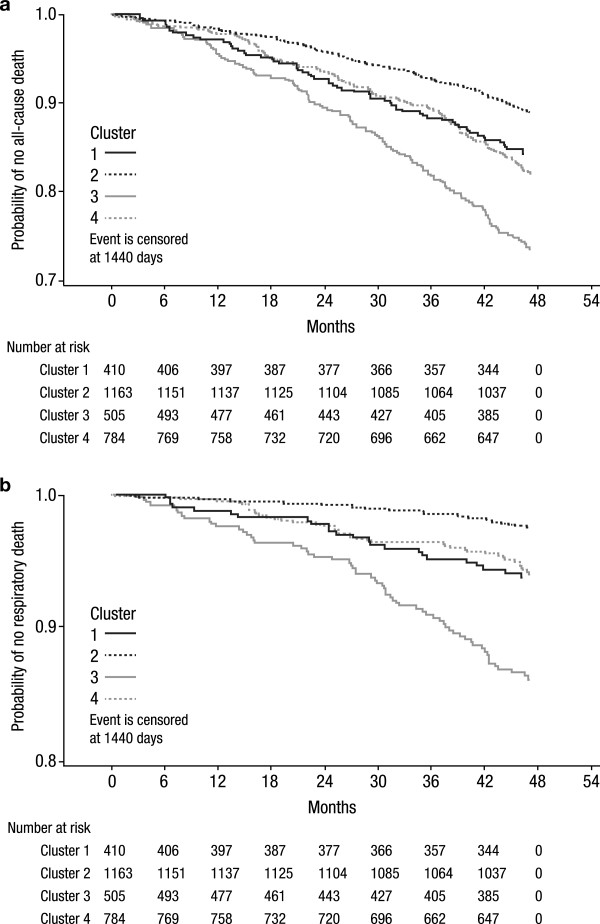
**Kaplan-Meier plots of mortality.** All-cause **(A)** and respiratory **(B)** mortality by cluster in the control group (n = 2862 patients) are shown.

**Figure 3:**
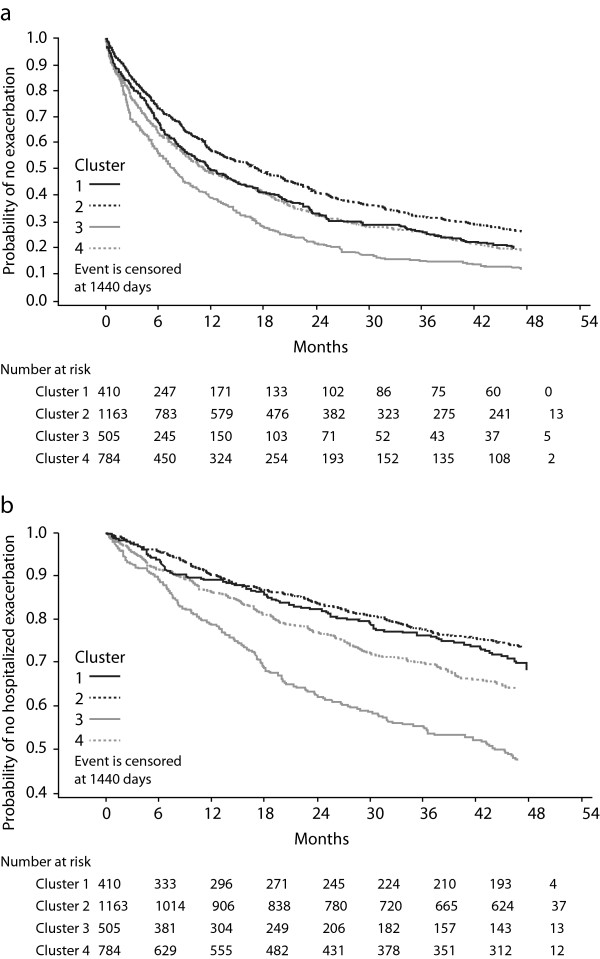
**Kaplan-Meier plots of exacerbations.** Time to first exacerbation **(A)** and severe exacerbation **(B)** by cluster in the control group (n = 2862 patients) are shown.

**Figure 4:**
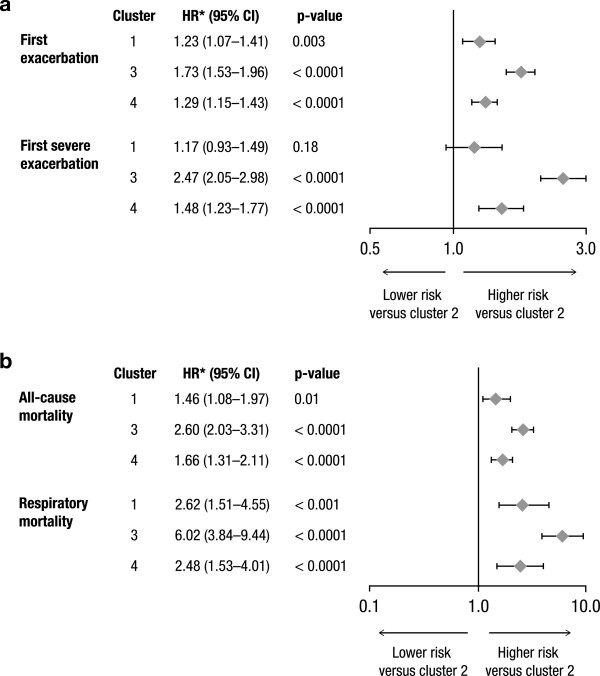
**Comparison of baseline risks of first exacerbation and first severe exacerbation (A), all-cause and respiratory mortality. (B)** by clusters in the control group (n = 2862 patients) are shown. CI = confidence interval, HR = hazard ratio.

The total number of deaths in the control group was 463 (16.1%). All-cause mortality rates were markedly different among clusters ranging from 11.1% in cluster 2 to 26.1% in cluster 3 (Table 2). Deaths related to respiratory causes occurred in 158 (5.5%) patients. Respiratory mortality was very low in cluster 2 (2.1%) and very high in cluster 3 (12.7%). Kaplan-Meier analyses of all-cause mortality and respiratory mortality by cluster are presented in Figure 2. Cox regression showed significant difference among clusters (p < 0.0001).Risk of exacerbations and severe exacerbations were markedly different among clusters. Patients in cluster 3 had the highest rates of exacerbations (1.21 ± 0.06 per patient/year) and hospitalizations (0.33 ± 0.03 per patient/year), whereas patients in cluster 2 had the lowest rates of exacerbations (0.69 ± 0.03 per patient/year) and hospitalizations (0.11 ± 0.01 per patient/year). Kaplan-Meier analyses of time to first exacerbation and to first severe exacerbation by cluster in the control group are presented in Figure 3. Cox regression showed significant difference among clusters (p < 0.0001).Cox analyses comparing baseline risks of exacerbations, severe exacerbations, all-cause and respiratory mortality by clusters in the control group are shown in Figure 4.

**Table 2:** Exacerbations, severe exacerbations (hospitalization), and mortality rates in the control group (N = 2862) by cluster

	**Cluster 1**	**Cluster 2**	**Cluster 3**	**Cluster 4**
	**n = 410**	**n = 1163**	**n = 505**	**n = 784**
**Exacerbations**				
Exacerbations, per patient-year (no.)				
Total	0.81 ± 0.05	0.69 ± 0.03	1.21 ± 0.06	0.91 ± 0.04
Leading to hospitalization	0.12 ± 0.02	0.11 ± 0.01	0.33 ± 0.03	0.17 ± 0.02
Time to first exacerbation (months*)				
Total	11.9 (10.1, 14.9)	17.3 (14.9, 20.0)	7.8 (6.6, 8.9)	11.2 (9.7, 13.9)
Leading to hospitalization	38.0 (29.9, 49.5)	43.8 (35.8, N/A)	14.6 (11.9, 17.3)	27.1 (22.8, 31.2)
Patients with exacerbation (n, %)				
Total	279 (68.0)	752 (64.7)	370 (73.3)	543 (69.3)
Leading to hospitalization	96 (23.4)	255 (21.9)	196 (38.8)	219 (27.9)
**Mortality**				
All cause, (n, %)	64 (15.6)	129 (11.1)	132 (26.1)	138 (17.6)
Respiratory, (n, %)	24 (5.9)	27 (2.3)	64 (12.7)	43 (5.5)

Data are mean SD or n (%); *First quartile, 95% CI.

*CI* = Confidence interval, *N/A* = not estimable, *SD* = Standard deviation.

### Effect of tiotropium on exacerbations and mortality by clusters

**Figure 5:**
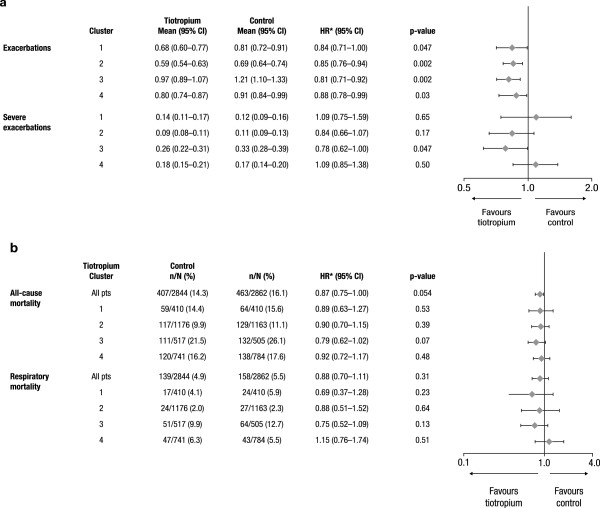
**Impact of tiotropium on the risk of exacerbations and severe exacerbations (A), all-cause and respiratory mortality. (B)** by clusters (n = 5706 patients) are shown. CI = confidence interval, HR = hazard ratio, pts = patients.

Because the clusters were defined by baseline factors, and baseline characteristics were comparable between treatment groups within each cluster, it is possible to compare treatment groups by cluster. Tiotropium significantly reduced exacerbations rates (Figure 5) and also increased time to first exacerbation in each cluster (not shown). Rates of severe exacerbations (leading to hospitalization) were significantly reduced in cluster 3 (p < 0.05), which had the highest rate of exacerbations leading to hospitalization, but not in other clusters.

When considering all patients (n = 5706), all-cause mortality was almost significantly reduced in the tiotropium versus the control group (hazard ratio [HR], 0.87; 95% confidence interval [CI], 0.75–1.00; p = 0.054). The effect was possibly driven by a clear tendency of tiotropium to reduce mortality in cluster 3 (HR, 0.79; 95% CI, 0.62–1.02; p = 0.07); whereas, in other clusters, the numerical reduction of mortality risk in tiotropium was mild (HR, 0.89–0.92) and not statistically significant (p > 0.35). Similarly, a trend towards reduction in respiratory mortality was observed in cluster 3 (HR, 0.75; 95% CI, 0.52–1.09; p *=* 0.13), but not in other clusters. The effect of tiotropium on all-cause and respiratory mortality by cluster is presented in Figure 5.

## Discussion

In the present study, we reasoned that classification of patients with COPD using a cluster analysis of multiple clinical variables previously associated with increased mortality may identify clusters of patients with variations in baseline risks of mortality. We found that risks and causes of mortality were markedly different among patients included in the UPLIFT® study. Cluster 2, which accounted for 41% of all patients, contained patients at low risk of mortality and very low risk of respiratory mortality; these patients were also at low risk of exacerbations and severe exacerbations. Compared with this low-risk cluster, risks of all-cause and respiratory mortality were 2.6- and six-fold higher in cluster 3, respectively, and were also increased in clusters 1 and 4, although to a lesser extent. Tiotropium reduced exacerbations in all clusters, and in particular reduced severe exacerbations in high-risk patients (cluster 3). Tiotropium was further suggested to reduce all-cause and respiratory mortality in high-risk patients (cluster 3), but not in other clusters. These findings underscore the potential impact of baseline risk stratification on the interpretation of the results of large clinical trials in patients with COPD.

The present analysis revealed that more than 40% of patients (cluster 2) included in this study had low risk of all-cause mortality and very low risk of respiratory mortality at the time of inclusion in the study. Patients in clusters 1 and 4 (who also represented more than 40% of all patients included) had higher baseline risk for all-cause mortality, but were also at low risk of respiratory mortality. Thus, more than 80% of patients included in the UPLIFT® study were at low baseline risk of respiratory mortality, which is in line with the risk of dying in the general population of those with COPD. When generating the hypothesis that tiotropium could reduce respiratory mortality, a reasonable hypothesis given its effect on FEV_1_ and exacerbations [4], the inclusion of this large number of low-risk patients may have resulted in missing the potential benefit of tiotropium in high-risk patients [7,8]. In support of this latter hypothesis, our analysis strongly suggested that tiotropium had a rather large impact on all-cause and respiratory mortality in high-risk patients (cluster 3), although the analysis was likely under-powered to show statistical significance.

To the best of our knowledge, no other study has evaluated the impact of heterogeneity in baseline risks and causes of mortality on the results of pharmacologic clinical trials in patients with COPD. In the past, analyses of potential efficacy of pharmacotherapy on mortality in patients with COPD have relied on conventional (and most often *post-hoc*) subgroup analyses of individual variables to examine variability in treatment effects among subgroups [3,5]. However, conventional subgroup analyses are inadequate to detect large and clinically important differences in treatment effect among patients when multiple factors determine risk [8]. This is typically the case for mortality risk in patients with COPD, which is determined by multiple factors including the level of FEV_1_ impairment, age, patient-centered outcomes (e.g. dyspnea, HRQoL), and comorbidities (e.g. malnutrition) [12,13]. For example, although cluster 3 contained 81% of GOLD Stage 3 and 4 patients (representing 54% and 22% of all GOLD Stage 3 and 4 patients, respectively), GOLD stage *per se* was not a significant covariate in the effect of tiotropium on all-cause mortality in a previously published analysis of the UPLIFT® study [5]. These data suggest that multi-dimensional assessment of baseline mortality risk is more appropriate to provide stratification of mortality risk in patients with COPD.

Our data strongly suggest that such stratification of clinical trials on baseline mortality risk is of utmost importance in studies assessing specifically mortality. Prognostic enrichment strategies, aimed at including subjects at high risk of mortality, may be especially useful in such studies [18]: because the appropriate sample size necessary to show a reduction in mortality rates will depend on effect size and the event rate in the placebo group, selecting a population at high risk of mortality would increase the likelihood of showing an effect of a drug, if there is one [18]. Prognostic enrichment may not increase the relative risk reduction (e.g. percent improvement in mortality), but will increase the absolute event counts, allowing for a smaller sample size [18]. Another advantage of this strategy will be to allow reduction in the duration of the study, presumably lowering study drop-off rates, a common problem in long-term clinical trials, and reducing the cost of the study.

In future studies, prognostic enrichment may rely on validated multivariate risk indices collected at study entry [12,13]. The BODE index, which is a predictor of all-cause and of respiratory mortality [13], may be particularly suited for selecting high-risk patients (and excluding low-risk patients) in clinical trials assessing the effect of therapy on mortality in patients with COPD. However, the BODE index requires a 6-minute walk test, which may be difficult to use in the screening of patients for large-scale multicenter studies. Alternative strategies for recruiting patients at high risk of mortality may eventually be considered. Based on the characteristics of high-risk subjects (cluster 3) in the present study, heavy current or ex-smokers with severe airflow limitation and impaired HRQoL (as measured by a high SGRQ total score) would be candidates for recruitment in these studies. Another possibility would be to recruit subjects who had experienced at least one hospitalization for COPD in recent years, as several studies have shown that 3-year mortality rates in these patients is approximately 50% [19,20]. Although the most effective enrichment strategy remains to be established, we suggest that future clinical trials assessing treatments aimed at reducing mortality in patients with COPD should include patients at high risk of mortality.

The present analysis was performed on patients with complete data for five variables, which were previously reported to predict mortality in patients with COPD and available in the UPLIFT® study. The UPLIFT® study was performed in 5993 patients, but 287 (4.8%) patients had missing data for these variables, leading to their exclusion from the analysis. Because these data were missing at random (not shown), as it is often the case in large clinical trials, this is unlikely to affect our conclusions significantly. Important predictors of mortality (e.g. dyspnea [13] and a history of severe exacerbations [21]) were not available for inclusion in the cluster analysis at study entry. Further, limited clinical data were available to characterize differences fully among the identified clusters. However, our analysis was validated by showing that these four clusters had marked heterogeneity in future outcomes, including exacerbations, hospitalizations, and all-cause and respiratory mortality. In the present study, risk of exacerbations was also heterogeneous among clusters, but tiotropium reduced exacerbations in all clusters of patients, confirming its positive impact on this outcome [4]. Consequences of the absence of risk stratification on the results of the UPLIFT® study were less important for exacerbations than for mortality. First, mortality was a relatively rare outcome that occurred in approximately 15% of patients, whereas exacerbations occurred in more than two thirds of patients over 4 years. Second, increases in all-cause and respiratory mortality risks in the highest- versus lowest-risk clusters (up to 2.6- and six-fold, respectively) were more important compared with those in exacerbation risks (fewer than two-fold). However, the impact of risk heterogeneity was also important for severe exacerbations leading to hospitalizations (a relatively rare event with substantial heterogeneity in risk), and tiotropium significantly reduced hospitalizations only in high-risk patients (cluster 3).

## Conclusion

These data suggest that appropriate patient selection is a critical component of clinical trial design to ensure that a sufficient number of patients that are at-risk of experiencing the outcome(s) of interest (e.g. death) are recruited in the study.

## Abbreviations

BMI: Body mass index; CI: Confidence interval; COPD: Chronic obstructive pulmonary disease; FEV_1_: Forced expiratory volume in 1 s; FVC: Forced vital capacity; GOLD: Global Initiative for Chronic Obstructive Lung Disease; HR: Hazard ratio; HRQoL: Health-related quality of life; IEC: Independent Ethics Committee; IRB: Institutional Review Board; SGRQ: St George’s Respiratory Questionnaire; TORCH: Towards a Revolution in COPD Health; UPLIFT®: Understanding Potential Long-term Impacts on Function with Tiotropium.

## Competing interests

In the past 5 years, Pierre-Régis Burgel has received fees for speaking, organising education or research, or consulting from Almirall, Nycomed-Takeda, AstraZeneca, Boehringer Ingelheim, Chiesi, GlaxoSmithKline, Novartis, Pfizer. Jean-Louis Paillasseur was full time employee of Clindatafirst and EFFI-STAT, CROs which received fees from Boehringer Ingelheim, Nycomed and AstraZeneca. Daniel Dusser has received fees for speaking, organising education or research, or consulting from Boehringer Ingelheim, Novartis, Pfizer, Chiesi, Dey Pharma and Nycomed. Nicolas Roche has received (i) fees for speaking, organising education or research, or consulting from Aerocrine, Almirall, Altana Pharma-Nycomed-Takeda, AstraZeneca, Boehringer Ingelheim, Chiesi, GlaxoSmithKline, MEDA, MSD-Chibret, Mundipharma, Novartis, Pfizer, Stallergenes, TEVA; (ii) research grants from Novartis, Nycomed, Boehringer Ingelheim and Pfizer. Yufeng Liu has received consulting fees from Boehringer-Ingelheim. Marc Decramer has received research grants or fees for consulting or speaking from Novartis, Nycomed, Boehringer Ingelheim, GlaxoSmithKline, Altana and AstraZeneca. Dacheng Liu, Armin Furtwaengler, and Norbert Metzdorf are full-time employees of Boehringer Ingelheim Pharma GmbH & Co KG.

## Authors’ contributions

PRB, JLP, DD, NR, and MD conceived and designed the paper; DL and YL performed the statistical analysis; PRB, JLP, DD, NR, AF, NM, and MD performed the analysis and interpretation. PRB, JLP, DD, NR, and MD helped to draft the manuscript for important intellectual content. All authors read and approved the final manuscript.
